# Cross-sectional and longitudinal comparison of health-related quality of life and mental well-being between persons with and without post COVID-19 condition

**DOI:** 10.3389/fepid.2023.1144162

**Published:** 2023-05-22

**Authors:** Emily Stella Scott, Erica I. Lubetkin, Mathieu F. Janssen, John Yfantopolous, Gouke J. Bonsel, Juanita A. Haagsma

**Affiliations:** ^1^ Department of Public Health, Erasmus MC, Rotterdam, Netherlands; ^2^ Department of Community Health and Social Medicine, CUNY School of Medicine, New York City, NY, United States; ^3^ Section Medical Psychology and Psychotherapy, Department of Psychiatry, Erasmus MC, Rotterdam, Netherlands; ^4^ Health Department of Economics, National and Kapodistrian University of Athens, Athens, Greece; ^5^ Department Scientific Support, EuroQol Research Foundation, Rotterdam, Netherlands

**Keywords:** post COVID-19 condition, COVID-19, health-related quality of life, EQ-5D-5L, mental well-being, longitudinal, chronic condition, healthy control group

## Abstract

**Background:**

Still little is known about the impact of post COVID-19 condition (PC) on health-related quality of life (HRQOL) and mental well-being. We compared participants with PC with three groups: an acute COVID-19 infection (AC) only, at least one chronic condition (CC) but no COVID-19, or no condition at all, healthy (PH). Between these disease groups, we also estimated and compared HRQOL and mental well-being change over time.

**Methods:**

Participants from six countries (Greece, Italy, Netherlands, Sweden, United Kingdom and United States) completed two web-based questionnaires (T1 = April–May 2020 and T2 = April–June 2022). Primary outcomes were HRQOL, measured by EQ-5D-5L and EQ VAS, and mental well-being (measured by World Health Organisation-Five (WHO-5) Well-Being Index, Patient Health Questionnaire (PHQ)-9 and General Anxiety Disorder (GAD)-7). All analyses were stratified by the disease groups.

**Results:**

In total, 4,999 participants filled out both surveys: 240 were in PC, 107 in AC, 1798 in CC and 2,854 in PH. At T2, the mean EQ-5D-5L index values for the PC, AC, CC and PH groups were 0.70, 0.73, 0.75 and 0.92 (*p* < .001), respectively. Mean EQ VAS scores were 66, 65, 68 and 81 (*p* < .001), respectively. Poor mental well-being, depression and anxiety mean values were highest in the PC group (47.7; 9.1; 7.4), followed by the AC group (51.1; 7.7; 5.7), CC group (56.1; 5.2; 4.2) and the PH group (65.6; 2.8; 2.5), respectively (*p* < .001 between groups). Over time, HRQOL deteriorated in all groups, apart from the PH group. We observed the largest deterioration in the CC (EQ-5D-5L index: Δ0.03, *p* < .001) and AC group (EQ VAS: Δ6.3, *p* < .001). For the mental well-being outcomes, deterioration for WHO-5 and PHQ-9 were largest in the AC group (Δ4.8, *p* = .016; Δ-1.3, *p* = .012). Rates for GAD-7 improved for the PH and CC groups (PH: Δ1.27, CC: Δ0.56, *p* < .001).

**Conclusions:**

In the cross-sectional analysis, participants with PC had the worst HRQOL and mental well-being compared to the other groups. In terms of change since the start of the COVID-19 pandemic, HRQOL and mental well-being deterioration was highest among AC participants and had a lower impact among PC participants, most likely due to pre-existing chronic disease.

## 1. Introduction

The long-term burden of the COVID-19 pandemic on population health is reflected in the 10%–20% of persons who report symptoms related to COVID-19 infection beyond three months (1). These long-term symptoms can typically include fatigue, shortness of breath, cognitive dysfunction and mental health problems. They are often described as an extension of the acute COVID-19 infection, although many of them are unlike acute phase symptoms, with some symptoms only developing sometime after the acute phase. Altogether, 20 or more different symptoms have been recognised as potentially long-term symptoms (2, 3). The World Health Organization (WHO) formally defined “post COVID-19 condition” as being present if there is continuation or development of new symptoms three months after the initial severe acute respiratory syndrome coronavirus 2 (SARS-CoV-2) infection, with symptoms lasting for at least two months, with no other explanation for the symptoms observed (4). So far, no limited set of condition-defining symptoms has been agreed on. Post COVID-19 condition may affect all domains of life, for example work, family relations, and social activities, thereby affecting health-related quality of life (HRQOL) (2) and mental well-being (5). Meta-analyses have shown that if post COVID-19 symptoms are present one month or more after the acute phase, HRQOL is negatively affected (6, 7). Similar evidence was found with longer follow-up (8). Mental well-being has been shown to be significantly impacted by post COVID-19 condition, too (9, 10). However, truly longitudinal evidence on the impact of post COVID-19 condition on HRQOL and mental well-being, i.e., evidence which is based on individual panel data collected before and after COVID-19 infection, is scarce. The commonly used recall data on HRQOL is lower than directly reported HRQOL (11). Longitudinal data would allow for a fair estimate of the impact of post COVID-19 condition and for determining prognostic modifiers once post COVID-19 condition arises. In the best case, preventable risk factors and effective care strategies are among these modifiers. In view of the specific pandemic effects on health, the use of healthy controls and of chronically diseased patients, with conditions sharing post COVID-19 condition symptoms, will further improve the detection and definition of post COVID-19 condition effects. As well as and not least, it would further improve the societal awareness of the conditions relevance to those affected.

In this study we use panel data from the longitudinal POPulation health impact of the CORoNavirus disease 2019 (COVID-19) pandemic (POPCORN) study. POPCORN is a large multi-national cohort-study set up in early 2020 to investigate the effect of the COVID-19 pandemic on HRQOL in the general population, with a special interest in the hypothesized deepening of health gaps across socio-economic and ethnic groups. The early timing of the POPCORN study has allowed us to collect data prior to the emergence of a large number of post COVID-19 condition-affected persons. The study encompassed a large set of outcome measures on HRQOL and mental well-being and the longitudinal structure allows for the comparison of health before and after acquiring post COVID-19 condition. As POPCORN comprises a general population cohort with detailed (co)morbidity information, comparisons between various sub-populations with and without post COVID-19 condition, but with other risks or morbidities are possible, allowing for our intended four-group (I-IV) comparison. Our aim is to estimate the HRQOL and mental well-being in persons with (I) post COVID-19 condition at T2 (April–June 2022) as well as compare their HRQOL and mental well-being with three other independent groups of participants, with either (II) an acute COVID-19 infection, (III) at least one chronic condition but no COVID-19, or (IV) no condition at all, assumed to be healthy. For these four groups, we also estimated the HRQOL and mental well-being at T1 (April–May 2020), and in a further comparison between the four groups, analysed the change in HRQOL and mental well-being from T1 to T2. We were additionally interested in who at T1 would go on to develop post COVID-19 condition at T2.

## 2. Materials and methods

### 2.1. Study design and population

This study is part of the POPCORN study. Data from the general population from six different countries, namely Greece, Italy, the Netherlands, Sweden, the United Kingdom and the United States (US), were collected via web-based surveys. All participants included in this study completed the survey at T1 (April–May 2020) and at T2 (April–June 2022). Further POPCORN studies have been published elsewhere (12).

### 2.2. Data collection procedure and consent

The surveys were distributed to participants by a market research agency (Dynata) at T1 and T2. This agency enlisted the participants from an existing large panel in these six countries. The study sample was designed to be representative by age, sex and educational level of the population in the respective country. Upon recruitment to the POPCORN project in 2020 (T1), participants were aged 18 to 75 years. To participate in these online surveys, participants already provided written informed consent upon registration when enrolling in the market research agencies' voluntary panels. As soon as participation in the survey had begun, the data capture system did not allow for any skipped or missed questions; therefore, this study has no missing values. An incentive in the form of cash or points (with a value between 3 and 6 Euro, depending on the participants country of residence) from the research agency was provided upon completion. Data were anonymised, and so the researchers had no direct access to individual participants.

### 2.3. Questionnaire

The questionnaires at T1 and T2 were close to identical, and included health outcome questions, medical risk factors (including e.g., smoking) and chronic conditions, socioeconomic determinant questions, COVID-19 related questions, demographic questions and questions on healthcare (access, use, etc.). The questionnaires were translated by human translators into the country's official language using computer-assisted translation software, followed by a translation back into English, except in the case of already available instruments with validated translated versions. Bilingual native speakers independently verified these translations. In Sweden, the T1 questionnaire was distributed between May 26 and June 1 in 2020, whereas in the remaining countries this was between April 22 and May 5 in 2020. In 2022 the questionnaires were distributed between 29 April and 25 June in all countries.

### 2.4. Primary outcome measures

Our primary outcome measures were HRQOL (measured by the EQ-5D-5L descriptive system, index values and EQ VAS) and mental well-being (measured by the World Health Organisation-Five (WHO-5) Well-Being Index, Patient Health Questionnaire (PHQ)-9 and General Anxiety Disorder (GAD)-7). The EQ-5D-5L descriptive system consists of a short set of five questions referring to the participants' self-reported health state today (13). It includes five dimensions, namely Mobility, Self-care, Usual activities, Pain/discomfort and Depression/anxiety. These dimensions can be rated on a 5-item scale from “no problems”, (“1”), to “extreme problems/unable to”, (“5”). The index values are derived from the EQ-5D-5L responses that have been weighted according to a value set, whereby the value set reflects societal preferences within a certain population (usually a country) of these health states. We used a single value set, the US value set (14), for each country, as this allows for cross-country comparisons. The EQ-5D-5L index values range from below 0 (“worse than death”) to 1 (“full health”), whereby the US value set ranges between −0.573 to 1. The EQ VAS (visual analogue scale) is the second part of the EQ-5D-5L instrument, whereby participants rate their overall health today on a scale from 0 (“The worst health you can imagine”) to 100 (“The best health you can imagine”).

The WHO-5 instrument measures subjective mental well-being referring to a period of the last two weeks (15). It is a generic scale without specific diagnostic specificity. It can be used across a wide range of study fields. It consists of five short positively-phrased questions about “feeling cheerful and in good spirits”, “feeling calm and relaxed”, “feeling active and vigorous”, “waking up feeling fresh and rested” and “daily life has been filled with things that interest me”, whereby the scale of six answers range between “all of the time” (“5”) to “at no time” (“0”). The WHO-5 index ranges from 0 (“worst imaginable well-being”) to 100 (“best imaginable well-being”), whereby these are calculated from the unweighted sum of the response scores, multiplied by 4. Using a cut-off score of ≤50 is generally recommendable when screening for clinical depression, and is the most widely used cut-off score across several different health study fields (15).

The PHQ-9 instrument assesses the presence of depressive disorders cf. DSM-IV; it also reflects depression severity, referring to a period of the last two weeks (16). The instrument consists of nine questions about how often the participant has been bothered by feelings or experiencing of “little interest or pleasure in doing things”, “down, depressed or hopeless”, “sleeping problems”, “tired or little energy”, “poor appetite or overeating”, “feeling bad about yourself”, “trouble concentrating”, “trouble moving or speaking slowly or being restless”, “thoughts of being better off dead or hurting self”. Answers are on a 4-item ordinal scale ranging between “not at all” to “nearly every day”. The PHQ-9 sum score ranges between 0 and 27, whereby depression severity is categorised into none (0–4), mild (5–9), moderate (10–14), moderately severe (15–19) and severe (20–27). The recommended cut-off score for screening for clinical depression is ≥10 (16, 17).

The GAD-7 instrument assesses the presence of generalised and other anxiety disorders and also reflects the level of anxiety in general, referring to a period of the last two weeks (18). The instrument consists of seven questions around “feeling nervous, anxious, or on edge”, “not being able to stop or control worrying”, “worrying too much about different things”, “trouble relaxing”, “being restless”, “becoming easily annoyed or irritable” and “feeling afraid as if something awful might happen”. Answers are on a 4-item ordinal scale ranging between “not at all” to “nearly every day”. The GAD-7 sum score ranges between 0 and 21, whereby anxiety severity is categorised into none (0–4), mild (5–9), moderate (10–14) and severe (15–21). The recommended cut-off score for screening for anxiety disorders is ≥8 (19, 20).

### 2.5. Respondent characteristics

Data on respondent characteristics included age, sex, highest attained education level, income, country of birth, COVID-19 vaccination status, chronic conditions, occupation and living situation. The highest attained education level was categorised into “high”, “middle” and “low” based on the International Standard Classification of Education (ISCED) 2011, levels ISCED 5–8, ISCED 3–4, ISCED 0–2, respectively. Data on the monthly household income from all sources after taxes was collected for each country in their respective currency, and categorised into three groups, namely “low” (lower 20% of the countries' populations income brackets), “middle” (middle 60%) and “high” (upper 20%) income. Country of birth was dichotomised to “native” and “non-native”, based on whether the country of birth was the participant's country of occupancy (either of the six countries) or not. Chronic conditions were dichotomised to “None” or “One or more”; participants were included in the latter category if any one or more of the following chronic conditions was selected: asthma, chronic bronchitis, lung emphysema, heart disease, consequences of a stroke, diabetes, chronic rheumatoid arthritis, severe back complaints/arthrosis of the back, painful/swollen joints of knee, hip or hands due to arthrosis, situation after knee/hip replacement, cancer, memory problems due to a disease, memory problems due to ageing, depression or anxiety disorder and an open text field for other chronic health complaints. Occupation information included employed (employee or self-employed), out of work (for >1 or <1 year), looking after others, student, retired and unable to work. Living situation was categorised into “Living alone” (living alone; living alone with one or more children), “Living with others” (living with a partner without children/with one or more children; living with my parents without children/with one or more children; living with my parents and partner with one or more children; living with roommates) and “Other” (other).

### 2.6. Disease status categorisation

For the purpose of this study, the four disease status groups were defined based on the T2 questionnaire data, namely (I) Post COVID-19 condition, (II) Acute COVID-19 infection, (III) Chronic condition(s) and (IV) Healthy, and are used throughout the study. Those participants in the first (post COVID-19 condition) group were defined by having a likely or confirmed COVID-19 infection in the past and indicating still suffering from symptoms; however, their infection did not occur within the last three months prior to when the questionnaire was sent out. Participants in the second (acute COVID-19 infection) group have the same criteria except that their infection occurred within the last three months prior to the questionnaire. These criteria are in accordance with the WHO post COVID-19 condition definition. Participants in the third [chronic condition(s)] group indicated suffering from one or more chronic condition(s), but do not suffer from post COVID-19 condition nor an acute COVID-19 infection. Participants in the fourth (healthy) group form the remainder of the study population.

We additionally defined disease status categories at T1, in order to investigate which health state persons developing post COVID-19 condition at T2 were transitioning from at T1. However, the questions around post COVID-19 condition at T1 differ to the improved questions at T2, due to the lack of knowledge at the time of the development of the questionnaire given this was an emerging condition. Therefore, an exclusive post COVID-19 condition group at T1 does not exist, but instead is combined with likely or confirmed acute COVID-19 infections. The definitions are outlined in further detail in Supplementary Table S1.

### 2.7. Statistical analyses

Descriptive analyses were performed for the respondent characteristics data as well as all the outcome variables (EQ-5D-5L index values, EQ VAS, WHO-5, PHQ-9 and GAD-7 sum scores). All analyses, including the longitudinal analyses, were carried out separately by disease status group at T2. For age-specific analyses, age was split by the median of the total study sample. To test for a difference in respondent characteristics across the disease groups at T2 as well as in the non-response analysis, we used the one-way ANOVA (for the continuous variable age), and the Fisher's exact and chi-square tests (for remaining categorical variables). A one-way ANOVA was applied to determine the difference in mean outcome between the disease status groups at T2. We included a multiple comparison post-hoc analysis test using the Bonferroni correction method. Detailed responses on the EQ-5D-5L dimensions and WHO-5, PHQ-9 and GAD-7 were graphically displayed through stacked bar charts, whereby the WHO-5, PHQ-9 and GAD-7 sum scores were dichotomised into “good” and “poor” so that participants with a score ≤50 for WHO-5, ≥10 for PHQ-9 and ≥8 for GAD-7 were considered as “poor”. This allowed for visual comparison of outcome patterns across the four disease groups. Change in disease status category was graphically displayed by a Sankey plot. We then determined the outcome indicator change between T1 and T2 by applying paired samples t-tests. The paired differences were calculated using the sum score ranges, thereby a positive (+ve) change in the mean from T1 to T2 is referred to as a “deterioration” in the case for the EQ-5D-5L index value, EQ VAS and WHO-5 score changes and as an “improvement” for the PHQ-9 and GAD-7 score changes. Finally, we described the association (clustering) between WHO-5, PHQ-9 and GAD-7 through Venn diagrams. A *p*-value of <.05 was required for statistical significance. Statistical analyses were carried out using IBM SPSS version 28.0.1.0, and figures were produced using Windows Excel (Bar charts and box & whisker plots) and R Studio (Sankey plots, Venn diagrams). For the Venn diagrams, the eulerr R package was used (21).

## 3. Results

### 3.1. Study population

Out of the 19 902 respondents from Greece, Italy, the Netherlands, Sweden, the United Kingdom and the US who completed the questionnaire at T1, 4 999 (response rate: 25%) also completed the questionnaire at T2. Responders at T2 were significantly different compared to non-responders in gender, age, educational level, country and chronic conditions (Supplementary Table S2). The response rate among countries varied between 20% among the Dutch and US respondents and 37% among Greek respondents. Table 1 shows the characteristics at T2 among the 4 999 respondents in total and by disease status. At T2, the median (IQR) age of all respondents was 55 (22). Slightly more than half of all respondents were female (52.5%), high-educated (50.7%) or without chronic conditions (60.1%).

**Table 1:** Characteristics of respondents by T2 disease status (*n* = 4,999).

Variable	Categories	Frequency (% of variable)	Cases of post COVID-19 condition [*n* (%)], *N* = 240	Cases of acute COVID-19 infection [*n* (%)], *N* = 107	Cases of chronic condition(s) [*n* (%)], *N* = 1,798	Healthy individuals [*n* (%)], *N* = 2,854	*p*-value
Gender	Male	2,372 (47.4)	100 (41.7)	48 (44.9)	828 (46.1)	1,396 (48.9)	.199
	Female	2,622 (52.5)	140 (58.3)	59 (55.1)	968 (53.8)	1,455 (51)	
	Other	5 (0.1)	*0*	*0*	2 (0.1)	3 (0.1)	
Age	Median (IQR)	55 (22)	49 (18)	52 (20)	60 (19)	52 (22)	<.001
	Mean (SD)	53.6 (13.7)	49.6 (12.9)	50.8 (12.9)	57.2 (13.2)	51.8 (13.6)	
Age	18–24	65 (1.3)	3 (1.3)	1 (0.9)	20 (1.1)	41 (1.4)	.000
	25–34	465 (9.3)	27 (11.3)	13 (12.1)	110 (6.1)	315 (11)	
	35–44	852 (17.0)	57 (23.8)	27 (25.2)	205 (11.4)	563 (19.7)	
	45–54	1,060 (21.2)	60 (25)	21 (19.6)	323 (18)	656 (23)	
	55–64	1,196 (23.9)	58 (24.2)	25 (23.4)	476 (26.5)	637 (22.3)	
	65–77	1,361 (27.2)	35 (14.6)	20 (18.7)	664 (36.9)	642 (22.5)	
Country	Greece	376 (7.5)	12 (5)	10 (9.3)	140 (7.8)	214 (7.5)	.000
	Italy	1,165 (23.3)	49 (20.4)	19 (17.8)	356 (19.8)	741 (26)	
	The Netherlands	644 (12.9)	34 (14.2)	19 (17.8)	272 (15.1)	319 (11.2)	
	Sweden	729 (14.6)	50 (20.8)	10 (9.3)	343 (19.1)	326 (11.4)	
	United Kingdom	873 (17.5)	52 (21.7)	30 (28)	287 (16)	504 (17.7)	
	United States	1,212 (24.2)	43 (17.9)	19 (17.8)	400 (22.2)	750 (26.3)	
Country of birth*	Native	4,719 (94.4)	229 (95.4)	102 (95.3)	1,700 (94.5)	2,688 (94.2)	.808
	Non-native	280 (5.6)	11 (4.6)	5 (4.7)	98 (5.5)	166 (5.8)	
Chronic condition(s)^£^	None	3,004 (60.1)	96 (40)	54 (50.5)	NA	2,854 (100)	.069
	One or more	1,995 (39.9)	144 (60)^$^	53 (49.5)	1,798 (100)	NA	
COVID-19 vaccination status	Yes	4,427 (88.6)	212 (88.3)	91 (85)	1,630 (90.7)	2,494 (87.4)	.005
	No	572 (11.4)	28 (11.7)	16 (15)	168 (9.3)	360 (12.6)	
Education level*	High	2,534 (50.7)	127 (52.9)	64 (59.8)	817 (45.4)	1,526 (53.3)	<.001
	Middle	1,973 (39.5)	88 (36.7)	37 (34.6)	764 (42.5)	1,084 (38)	
	Low	492 (9.8)	25 (10.4)	6 (5.6)	217 (12.1)	244 (8.5)	
Income level	High	782 (15.6)	40 (16.7)	14 (13.1)	247 (13.7)	481 (16.9)	.005
	Middle	2,867 (57.4)	131 (54.6)	69 (64.5)	1,015 (56.5)	1,652 (57.9)	
	Low	1,350 (27)	69 (28.7)	24 (22.4)	536 (29.8)	721 (25.3)	
Occupation	Employed (employee)	2,358 (47.2)	124 (51.7)	58 (54.2)	628 (34.9)	1,548 (54.2)	.000
	Employed (self-employed)	395 (7.9)	20 (8.3)	8 (7.5)	117 (6.5)	250 (8.8)	
	Out of work for >1 year	327 (6.5)	22 (9.2)	4 (3.7)	119 (6.6)	182 (6.4)	
	Out of work for <1 year	71 (1.4)	5 (2.1)	*0*	30 (1.7)	36 (1.3)	
	Looking after others	204 (4.1)	9 (3.8)	2 (1.9)	60 (3.3)	133 (4.7)	
	Student	66 (1.3)	3 (1.3)	1 (0.9)	23 (1.3)	39 (1.4)	
	Retired	1,369 (27.4)	38 (15.8)	23 (21.5)	671 (37.3)	637 (22.3)	
	Unable to work	209 (4.2)	19 (7.9)	11 (10.3)	150 (8.3)	29 (1)	
Living situation	Living alone	1,464 (29.3)	60 (25)	39 (36.4)	601 (33.4)	764 (26.8)	<.001
	Living with others	3,506 (70.1)	178 (74.2)	68 (63.6)	1,182 (65.7)	2,078 (72.8)	
	Other	29 (0.6)	2 (0.8)	*0*	15 (0.8)	12 (0.4)	

^*^ Variable data is based on data retrieved at T1 POPCORN questionnaire and not T2.

^$^ None of the chronic condition(s) listed by individuals in the post COVID-19 condition group were post COVID-19 condition or any similar name.

^£^ The chi-square test was only applied to the acute COVID-19 infection and post COVID-19 condition groups. The *p*-value corresponds to the tests of independence between disease status groups.

### 3.2. Description of respondents according to disease status

At T2, 240 (5%) were considered to have post COVID-19 condition, 107 (2%) had an acute COVID-19 infection, 1,798 (36%) had one or more chronic condition(s) and 2,854 (57%) participants were presumably healthy (Table 1 and Supplementary Table S1).

**Figure 1:**
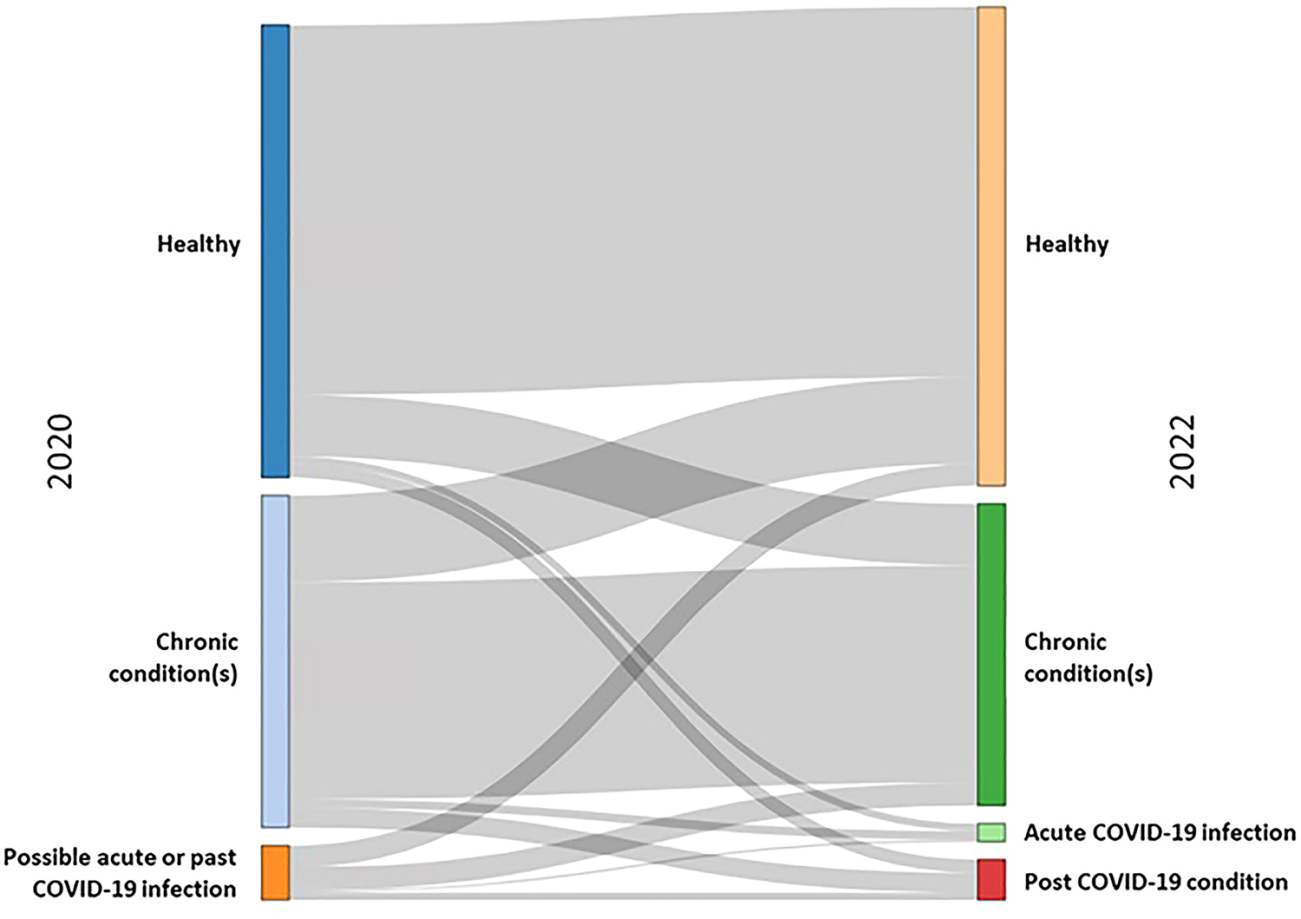
Transitions between disease status groups in 2020 (T1) [healthy, chronic condition(s), possible acute or past COVID-19 infection] to 2022 (T2) [healthy, chronic condition(s), acute COVID-19 infection and post COVID-19 condition].

In Table 1, the age distribution differed between the disease groups, with the median (IQR) age in the post COVID-19 condition group [49 (18)] being the lowest and highest in the chronic condition(s) group [60 (19)] compared to the other groups. Country, COVID-19 vaccination status, educational level, income level, occupation and living situation also differed across the disease status groups, while country of birth and chronic condition(s) (between only the acute COVID-19 infection and post COVID-19 condition groups), did not. Notably still, 60% of persons with post COVID-19 condition had one or more chronic condition(s) at T2, compared to 39.9% in the total sample population (Table 1). In analysing the transition in disease status from T1 to T2 (Figure 1), the largest proportion of participants with post COVID-19 condition at T2 was previously in the chronic condition(s) group at T1 (*n* = 116, 48.3%), followed by 80 (33.3%) from the healthy group and 44 (18.3%) from the possible acute or past COVID-19 infection group. Therefore, when having one or more chronic condition(s) at T1, the likelihood of having post COVID-19 condition was 5.9%, compared to 3.0% when being healthy. The group of individuals who had chronic condition(s) at T1 and became healthy at T2 were separately analysed due to the large number transitioning between these states (Supplementary Figure S1).

### 3.3. Primary outcomes at T2

**Figure 2:**
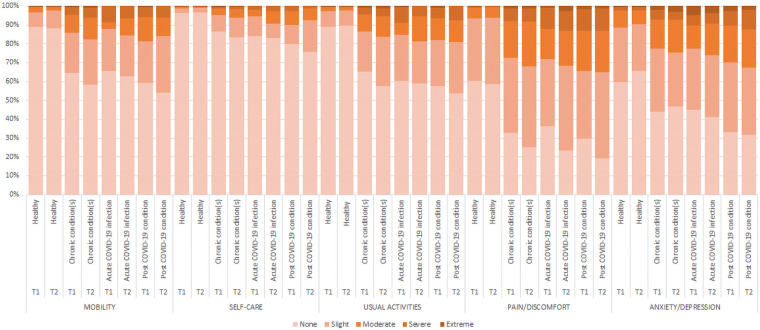
Percentage of respondents per level per EQ-5D-5L dimension in 2020 (T1) and 2022 (T2), by T2 disease status.

Figure 2 shows that healthy participants reported the lowest rates of any problems (slight to extreme problems/unable to) in all EQ-5D-5L dimensions, whereby the post COVID-19 condition group reported the highest rates of any problems, compared to all other groups. The T2 EQ-5D-5L index value (Figure 4A) was lowest among the post COVID-19 condition group [mean (SD) = 0.70 (0.29)], followed by the acute COVID-19 infection group [0.73 (0.29)], the chronic condition(s) group [0.75 (0.25)], and highest in the healthy group [0.92 (0.12)]. For the EQ VAS (Figure 4B), the lowest HRQOL was reported in the acute COVID-19 infection group [mean (SD) = 65.1 (20.1)], followed by the post COVID-19 condition group [65.7 (21.0)], the chronic condition(s) group [68.4 (19.8)], and highest in the healthy group [80.8 (14.3)]. The pattern in the EQ-5D-5L respondent rates per dimension between disease groups is largely maintained across the two age groups, 18–54 and 55–77, compared to the total study sample at T2 (Supplementary Figures S2A,B).

**Figure 3:**
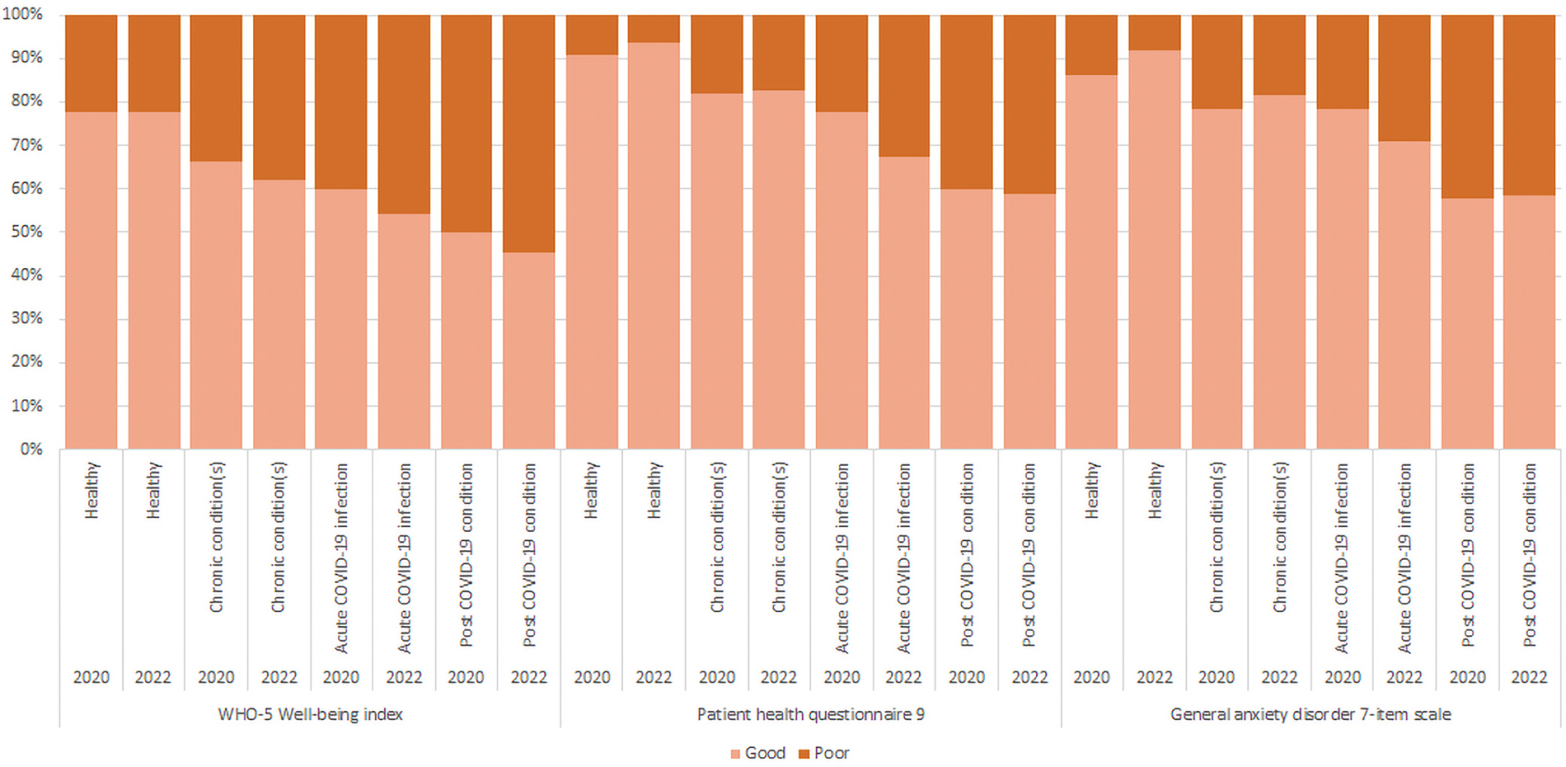
Percentage of respondents with poor mental health according to the WHO-5, PHQ-9 and GAD-7 in 2020 (T1) and 2022 (T2), by T2 disease status. The WHO-5 well-being index measures overall mental well-being, whereby poor mental well-being ranges from 0 to 50 points and good mental well-being from 51 to 100 points. The Patient health questionnaire 9 (PHQ-9) measures depression, whereby depression (i.e. “poor PHQ-9”) includes moderate, moderately severe and severe depression, which ranges from 10 to 27. The General anxiety disorder 7-item scale (GAD-7) measures anxiety, whereby having anxiety (i.e. “poor GAD-7”) includes mild, moderate and severe anxiety from the raw score (ranging from 8 to 21), and no anxiety ranges between 0 and 7.

**Figure 4:**
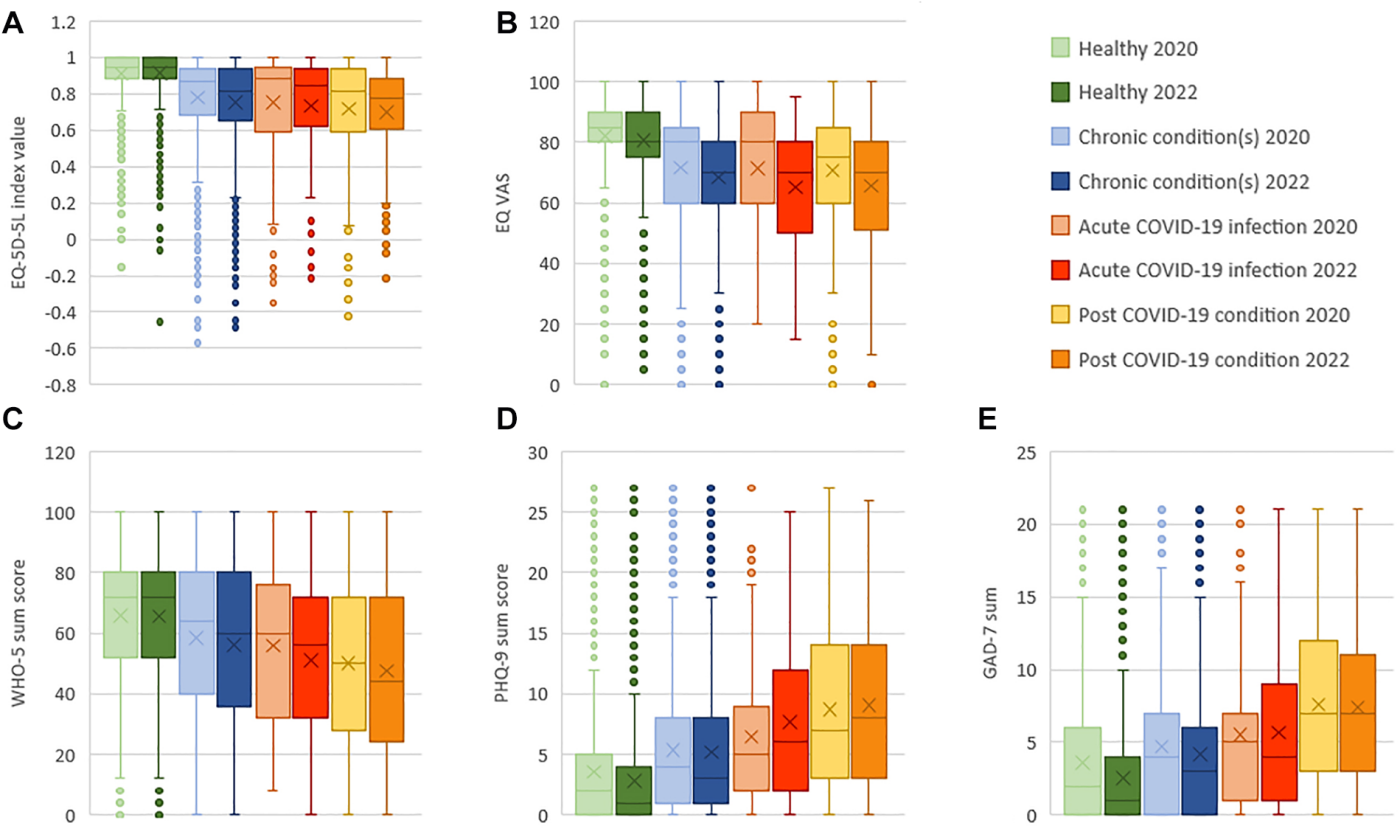
(**A–E**): Mean and median EQ-5D-5L index value (**A**), EQ VAS score (**B**), WHO-5 sum score (**C**), PHQ-9 sum score (**D**) and GAD-7 sum score (**E**) in 2020 (T1) and 2022 (T2), by T2 disease status. The X denotes the mean, the line in the box the median, the box is the interquartile range, and the whiskers are the minimum and maximum points with outliers removed and depicted as dots. For the (**A**) EQ-5D-5L index values we used the United States value set. EQ-5D-5L index values range from less than 0 (worse than death) to 1. The (**B**) EQ VAS (Visual analogue scale) ranges from 0 (worst self-rated health) to 100 (best self-rated health). The (**C**) WHO-5 sum score (WHO-5 Well-being index) ranges from 0 (worst imaginable well-being) to 100 (best imaginable well-being). The (**D**) PHQ-9 sum score (Patient health questionnaire 9) ranges from 0 to 27 (Mild: 5–9, Moderate: 10–14, Moderately severe: 15–19, Severe: 20–27). The (**E**) GAD-7 sum score (General anxiety disorder 7-item scale) ranges from 0 to 21 (Mild: 5–9, Moderate: 10–14, Severe: 15–21).

Rates for poor mental well-being, depression and anxiety disorder at T2 (Figure 3) were highest in the post COVID-19 condition group followed by the acute COVID-19 infection group, the chronic condition(s) group, and lowest in the healthy group. In Figures 4C–E, the mean (SD) scores are lowest in the post COVID-19 condition group [WHO-5: 47.7 (26.7); PHQ-9: 9.1 (6.7); GAD-7: 7.4 (5.8)], followed by the acute COVID-19 infection group [WHO-5: 51.1 (25.6); PHQ-9: 7.7 (6.5); GAD-7: 5.7 (5.7)], the chronic condition(s) group [WHO-5: 56.1 (25.9); PHQ-9: 5.2 (5.6); GAD-7: 4.2 (4.9)], and highest for the healthy group [WHO-5: 65.6 (23.2); PHQ-9: 2.8 (4.0); GAD-7: 2.5 (3.8)]. Comparing the two age groups, 18–54 and 55–77, the younger population had overall higher rates of poor mental well-being (WHO-5, PHQ-9 and GAD-7) at T2 (Supplementary Figures S3A,B). The difference in means between the disease status groups in all the HRQOL and mental well-being outcomes was significant (*p* < .001) (see Supplementary Table S3 for Figures 4A–E values).

In the multiple comparison post-hoc analysis test across disease status groups, the mean difference between the post COVID-19 condition group was statistically significantly different (*p* < .001) compared to the healthy group (EQ-5D-5L index: mean difference = .220; EQ VAS: 15.1; WHO-5: 17.9; PHQ-9: −6.3 GAD-7: −4.9), the chronic condition(s) group (EQ-5D-5L index:.05; WHO-5: 8.4; PHQ-9: −3.9; GAD-7: −3.3) and the acute COVID-19 infection group (GAD-7: −1.7, *p* = .004) (Supplementary Table S4).

### 3.4. Change (T1 to T2) in primary outcomes

From T1 to T2, rates of any problems (slight to extreme problems/unable to) deteriorated in all EQ-5D-5L dimensions among all disease status groups, apart from the healthy group who improved slightly in the self-care, usual activities and anxiety/depression dimensions and the chronic condition(s) group in the anxiety/depression dimension (Figure 2). From T1 to T2, HRQOL deteriorated in all four groups, except for the healthy group (EQ-5D-5L index: Δ0.005, *p* = .035) (Table 2 and Supplementary Tables S3). We observed the largest deterioration in the EQ-5D-5L index value in the chronic condition(s) group (EQ-5D-5L index: Δ0.03, *p* < .001). The largest deterioration in EQ VAS was observed in the acute COVID-19 infection group (EQ VAS: Δ6.3, *p* < .001) followed by the post COVID-19 condition group (EQ VAS: Δ4.96, *p* < .001).

**Table 2:** Paired samples t-test for the change between T1 and T2, by disease status.

Disease status		Paired Differences					*t*	*df*	Sig. (2-tailed)
		Mean	Std. Dev.	Std. Error Mean	95% confidence interval of the difference				
					Lower	Upper			
Healthy	EQ-5D-5L index value at T1 and T2^1^	−0.005*	0.12	0.00	−0.01	0.00	−2.1	2,853	0.035
	EQ VAS score at T1 and T2^2^	1.402*	12.72	0.24	0.94	1.87	5.9	2,853	<.001
	WHO-5 sum score at T1 and T2^3^	0.408	19.89	0.37	−0.32	1.14	1.1	2,853	0.273
	PHQ-9 sum score at T1 and T2^4^	0.754*	3.72	0.07	0.62	0.89	10.8	2,853	<.001
	GAD-7 sum score at T1 and T2^5^	1.272*	3.68	0.07	0.97	1.24	16.0	2,853	<.001
Chronic condition(s)	EQ-5D-5L index value at T1 and T2^1^	0.027*	0.17	0.00	0.02	0.04	6.8	1,797	<.001
	EQ VAS score at T1 and T2^2^	3.226*	16.72	0.39	2.45	4.00	8.2	1,797	<.001
	WHO-5 sum score at T1 and T2^3^	2.516*	20.14	0.48	1.58	3.45	5.3	1,797	<.001
	PHQ-9 sum score at T1 and T2^4^	0.182	4.34	0.10	−0.02	0.38	1.8	1,797	0.075
	GAD-7 sum score at T1 and T2^5^	0.561*	3.96	0.09	0.38	0.74	6.0	1,797	<.001
Acute COVID-19 infection	EQ-5D-5L index value at T1 and T2^1^	0.018	0.18	0.02	−0.02	0.05	1.1	106	0.295
	EQ VAS score at T1 and T2^2^	6.327*	17.78	1.72	2.92	9.74	3.7	106	<.001
	WHO-5 sum score at T1 and T2^3^	4.785*	20.26	1.96	0.90	8.67	2.4	106	0.016
	PHQ-9 sum score at T1 and T2^4^	−1.252*	5.07	0.49	−2.22	−0.28	−2.6	106	0.012
	GAD-7 sum score at T1 and T2^5^	−0.168	4.30	0.42	−0.99	0.66	−0.4	106	0.687
Post COVID-19 condition	EQ-5D-5L index value at T1 and T2^1^	0.023	0.21	0.01	−0.00	0.05	1.7	239	0.099
	EQ VAS score at T1 and T2^2^	4.963*	15.09	0.97	3.04	6.88	5.1	239	<.001
	WHO-5 sum score at T1 and T2^3^	2.567	23.34	1.51	−0.40	5.53	1.7	239	0.09
	PHQ-9 sum score at T1 and T2^4^	−0.388	5.76	0.37	−1.12	0.35	−1.0	239	0.298
	GAD-7 sum score at T1 and T2^5^	0.204	5.42	0.35	−0.49	0.89	0.6	239	0.56

A positive (+ve) difference in the mean from T1 to T2 is a “deterioration” in the case of the EQ-5D-5L index value, EQ VAS and WHO-5 score changes, and as an “improvement” for the PHQ-9 and GAD-7 score changes.

^1^ Pair 1 (EQ-5D-5L index value at T1 and T2).

^2^ Pair 2 (EQ VAS score at T1 and T2).

^3^ Pair 3 (WHO-5 sum score at T1 and T2).

^4^ Pair 4 (PHQ-9 sum score at T1 and T2).

^5^ Pair 5 (GAD-7 sum score at T1 and T2).

^*^ The mean difference is significant at the 0.05 level.

From T1 to T2, rates in poor mental well-being measured with WHO-5 remained stable in the healthy group and deteriorated in the other disease status groups (Figure 3), whereby this was statistically significant in the chronic condition(s) group (Δ2.5, *p* < .001) and in the acute COVID-19 infection group (Δ4.8, *p* = .016) (Table 2). The change in PHQ-9 rates for depression were a mix of improvement in the healthy group (Δ0.8, *p* < .001) and deterioration in the acute COVID-19 infection group (Δ-1.3, *p* = .012) (Table 2). The GAD-7 rates for anxiety improved from T1 to T2 in all groups except for the acute COVID-19 infection group (Figure 3), whereby this was statistically significant in the healthy participants (Δ1.3, *p* < .001) and the chronic condition(s) group (Δ0.6, *p* < .001) (Table 2). This pattern is largely maintained across the two age groups, 18–54 and 55–77, with only slight differences in rates such as in the post COVID-19 condition group for anxiety improving in the 18–54 age group compared to deteriorating in the 55–77 age group (Supplementary Figures S3A,B).

### 3.5. Overlap in mental well-being outcomes

**Figure 5:**
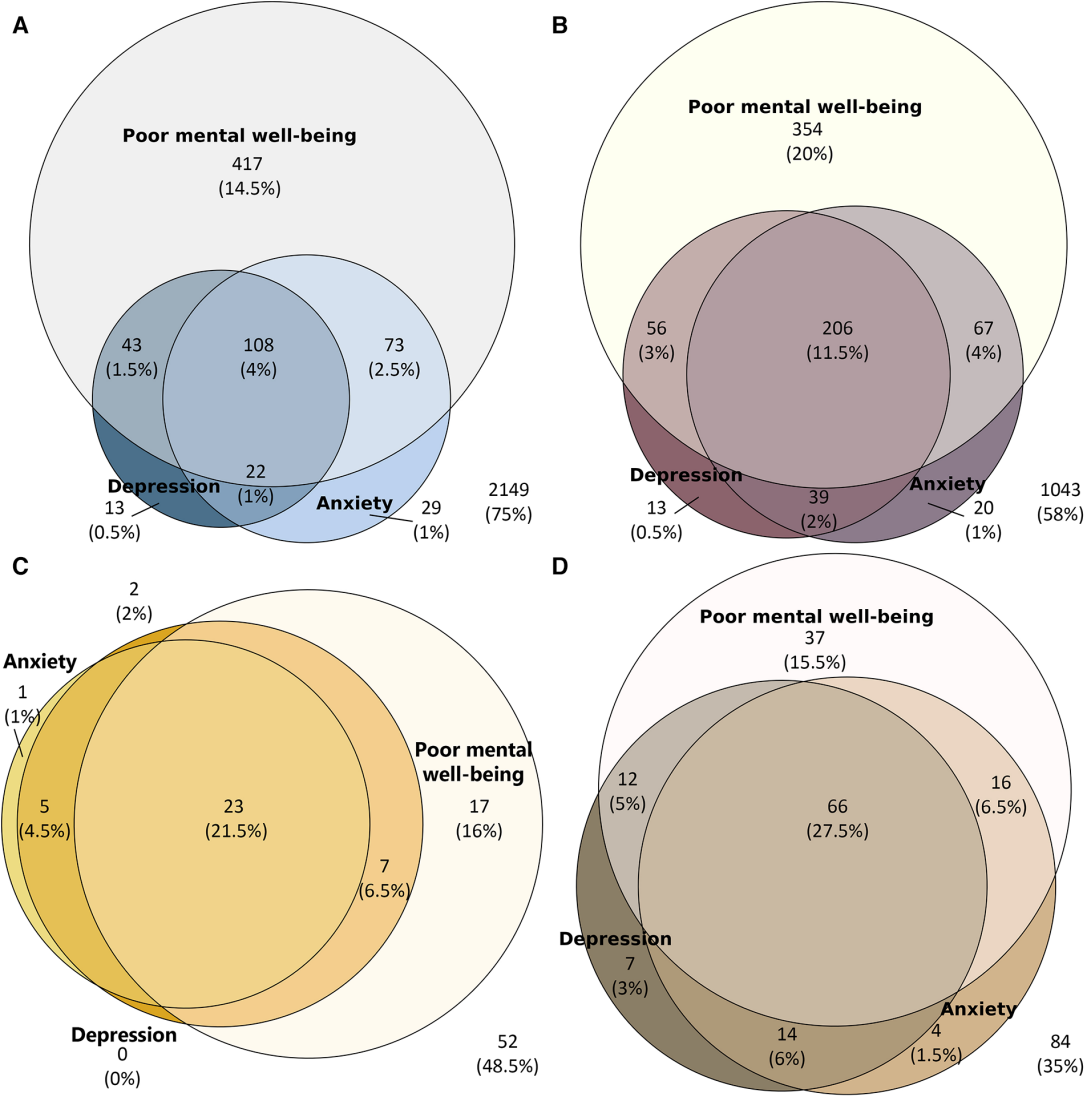
Overlap of poor mental well-being (WHO-5), depression (PHQ-9) and anxiety (GAD-7) at T2 in (A) healthy respondents (n=2854), (B) chronic condition(s) respondents (n = 1798), (C) acute COVID-19 infection respondents (n = 107) and (D) post COVID-19 condition respondents (n = 240).

Figures 5A–D present the co-occurrence of poor mental well-being, depression and anxiety for each disease status group. Healthy participants presented the largest group of unaffected respondents [2,149 (75%)] and the least overlap of all three outcomes [108 (4%)], in contrast to the post COVID-19 condition group who had the lowest number of unaffected respondents 84 (35%) and the most overlap [66 (27.5%)]. In the acute COVID-19 infection group, 23 (21.5%) respondents reported poor mental well-being on all outcomes and 206 (11.5%) respondents from the chronic condition(s) group. Correlations between HRQOL and mental health outcomes are presented in Supplementary Table S5, whereby all were positively correlated and significant.

## 4. Discussion

### 4.1. Key findings and comparative studies

Our study showed that participants with post COVID-19 condition overall had a higher prevalence of any problems on the EQ-5D-5L descriptive system in terms of mobility, self-care, usual activities, pain/discomfort and anxiety/depression, compared to the other disease groups. Furthermore, participants with post COVID-19 condition exhibited the worst HRQOL (mean and median EQ-5D-5L and EQ VAS) as compared to the other three groups, apart from the acute COVID-19 infection group reporting a slightly worse EQ VAS outcome. The prevalence of any problems on the EQ-5D-5L descriptive system observed in our post COVID-19 condition group was higher than the prevalence reported by Malik et al. in their meta-analysis of 12 studies on HRQOL of post COVID-19 condition patients (6). In addition, the mean EQ VAS score of respondents with post COVID-19 condition was lower than that of the meta-analysis (6). This discrepancy in findings may be due to a difference in the definition of post COVID-19 condition that was used. In the study of Malik et al., the case definition did not include a restriction on the duration since the acute COVID-19 infection, whereas in our study a respondent was identified as having post COVID-19 condition if symptoms were still occurring 3 months or longer since after the acute COVID-19 infection. Moreover, the discrepancy in HRQOL may further be explained by the relatively high proportion of respondents having co-morbid condition(s) in our post COVID-19 condition group (60% prevalence), compared to the studies included in the meta-analysis. We doubt whether this is a valid comparison, as the source studies of the meta-analysis did not include a similar extensive risk factor and comorbidity list with complete checking. We therefore attribute the higher HRQOL impact in our study mainly to a selection-through-definition effect.

As in the example above, few studies have used a restrictive minimum symptom duration of 3 months post-acute infection as corresponds to the WHO post COVID-19 condition case definition. In addition, even fewer studies have sought to compare this with a healthy group or other comparative (sub)population. One study included a comparison of HRQOL (median EQ-5D-5L and EQ VAS) between persons with post COVID-19 condition to persons with an acute COVID-19 infection with symptoms not lasting beyond 12 weeks or no symptoms at all, without a healthy control group. The former group's HRQOL (in both measures), were statistically significantly worse than the latter group's (22). This comparison holds true in our study, apart from the mean EQ VAS score being slightly worse in the acute COVID-19 infection group, although this was not statistically significant. This may be due to a difference in the acute COVID-19 infection group definitions, which in our definition does not include non-symptomatic infections. In a further study, HRQOL measured with the SF-36 was compared between a group of patients considered to have “long COVID” and a healthy control group consisting of young people at universities, however these participants were solely presumed to be healthy. Not surprisingly, the comparison demonstrated a significantly worse HRQOL in the “long COVID” group (23). Another study comparing a group of post-COVID-19 infected persons with persisting symptoms to a normative general population, observed better HRQOL in both the EQ-5D-3l and EQ VAS in the latter group (24). A detailed comparison of their mean scores to our data, showed even worse HRQOL in the post-COVID-19 infected persons compared to our post COVID-19 condition sample (EQ-5D-3l index value = 0.57 vs. EQ-5D-5L index value = 0.70; EQ VAS = 56.6 vs. 65.7, respectively). An obvious explanation for the comparatively low HRQOL scores, is that the post-COVID-19 infected patients were not specifically defined, and by use of a convenience sample, leading to selection bias.

Similar to HRQOL, poor mental well-being was also most prevalent in post COVID-19 condition participants compared to the other groups in our study. In a large matched cohort study including 145,184 adults with post COVID-19 condition and 723,165 matched controls that were not infected with SARS-CoV-2, the incidence rate ratio for mental health problems, which include depression and anxiety disorders among others, was found to be 1.27 (95% CI: 1.25–1.29) in the former group compared to the matched controls (25). Though we did not measure incidence, the higher prevalence of poor mental well-being in the post COVID-19 condition group compared to the remaining groups can be an indication of an increased incidence in this group relative to the others. Similarly, in the few studies where a control group was used, depression and anxiety were more frequent among those with persisting symptoms after a COVID-19 infection (however duration of persisting symptoms is not defined) compared to the healthy participants (5). Furthermore, rather than evaluating self-reported health questionnaires, a further study sought to evaluate diagnoses of psychiatric disorders, which include psychiatric illness, mood and anxiety disorder, in COVID-19 survivors (not specifically post COVID-19 condition). In this study, it was found that the COVID-19 survivors had a roughly doubled risk of having a newly diagnosed psychiatric disorder 14 to 90 days after a COVID-19 infection compared to matched cohorts that had been diagnosed with another similar health event (such as influenza, respiratory tract infection, skin infection or fracture of a large bone) (26). Though the COVID-19 survivors are not an entirely comparable group to those with post COVID-19 condition, a proportion of the survivors will go on to develop post COVID-19 condition, and the results alone indicate the possible long-term effects that are encountered in post COVID-19 condition. Together, these corroborate our findings that persons with post COVID-19 condition have significantly poorer mental health compared to our remaining groups.

When analysing the longitudinal data over the two years, rates of any problems in HRQOL increased in all groups apart from a few exceptions in healthy and chronic condition(s) participants, with the post COVID-19 condition group already having a higher prevalence of any problems in HRQOL compared to the remaining groups in 2020. Our study's finding that an increased risk in acquiring post COVID-19 condition is associated with prior presence of one or more chronic conditions, corresponds to previous studies that detected a 26% increased risk for individuals with pre-existing comorbidities (27). Furthermore, a strong predictor for post COVID-19 condition was overall burden of comorbidity in the study by Förster et al. (22). Similarly, in a study where information on pre-existing chronic conditions was systematically gathered, the authors found that having one, 2–3 and 4 or more chronic conditions inferred between 23% - 121% and 16% - 90% increased risk among those not having recovered at all or having only partially recovered, respectively, 12 and 18 months after a symptomatic COVID-19 infection (8).

Provided the overall deterioration of HRQOL in this group from 2020 to 2022, we expected to see a similar pattern in all three mental well-being outcomes. However, an overall decrease in the rate of anxiety in the majority of the disease groups as well as a significant improvement in the mean anxiety levels in the healthy and chronic condition(s) participants was detected. This could be due to a global decrease in anxiety due to the adaptation to the novelty of the COVID-19 pandemic and the morbidity and mortality associated with it. In the US, the National Center for Health Statistics estimated the prevalence of symptoms of anxiety disorder, which has also been steadily declining from April 2020 (32.8%) to 2021 (30.5%) to the end of 2022 (28.8%) (28). Moreover, the decrease in rates of anxiety may be due to the alleviation of governmentally induced restrictions or lockdowns in 2022 in the six included countries, as previous findings of the POPCORN study showed that the stringency of government response is associated with worse mental well-being (12).

### 4.2. Strengths and limitations

Strengths of our study are the longitudinal study design, which allow for the comparison of HRQOL and mental well-being from the beginning of the COVID-19 pandemic in 2020 to later phases of the pandemic, while also enabling the determination of the impact of post COVID-19 condition on HRQOL and mental well-being without having to rely on recall data. Another strength includes the comparison between persons with post COVID-19 condition to three further defined and mutually exclusive health states, which has been lacking so far in the literature. However, the accuracy of the post COVID-19 condition group cannot be guaranteed given the self-reported nature of the questionnaire, and the inclusion of “probable” COVID-19 infections in this group. Furthermore, compared to the chronic condition(s) group, we can speculate that the post COVID-19 condition group had worse HRQOL and mental well-being at T2 and a greater deterioration in HRQOL from T1 to T2 due to acquiring post COVID-19 condition and not due to their co-morbidities. This is because the chronic condition(s) group had overall better HRQOL and mental well-being at T2 and a smaller deterioration in HRQOL over time. However, we are very cautious with this interpretation because we did not test for differences between the two groups in the number and types of chronic conditions, as well as other indicators that could influence the outcomes. In addition, it is important to bear in mind that chronic conditions were self-reported and an option for “other” chronic conditions was available; therefore, conditions were not necessarily verified by health practitioners and were highly diverse and subjective. In all, this challenges the generalisability to the post COVID-19 condition population. Despite this limitation, contrarily to other studies where a convenience sample or a specific post COVID-19 condition population were obtained, e.g., that were hospitalised, we used a general population sample. Furthermore, respondents who did not complete the questionnaire at T2 were significantly younger and more often reported having chronic conditions, though this may have been largely mitigated given the large sample size. Taking into account the higher risk of developing post COVID-19 condition among those with chronic conditions, this may have led to a higher attrition of those with post COVID-19 condition at T2.

Moreover, the non-response analysis could not be applied to the disease status categorisation due to the T1 COVID-19 questions not matching the improved version at T2, given our lack of knowledge of post COVID-19 condition at the time of the production of the questionnaire, as well as the limited COVID-19 testing capacities. This has made the comparison between disease status classification less accurate and made loss to follow up difficult to detect between the disease status groups. On the other hand, the timing of the first wave data collection was close to the start of the pandemic, making it very unlikely to capture many people with post COVID-19 condition at T1.

Some recommendations include the concerted use of the WHO definition of post COVID-19 condition, to ease comparability between epidemiological research, as well as the use of control groups including healthy and with chronic condition(s), as this may allow for the identification of commonalities, a clearer condition definition, and help identify treatment options.

### 4.3. Conclusions

We conclude that participants with post-COVID condition had the worst HRQOL and mental well-being compared to the other three groups. In terms of change since the start of the COVID-19 pandemic, HRQOL and mental well-being deterioration was highest among the participants with an acute COVID-19 infection and had a lower impact among participants with post-COVID condition, most likely due to pre-existing chronic disease.

## Data Availability

The original contributions presented in the study are included in the article/**Supplementary Materials**, further inquiries can be directed to the corresponding author/s.
