# Supplementary material for: Cross-sectional and longitudinal comparison of health-related quality of life and mental well-being between persons with and without post COVID-19 condition
Source: Front Epidemiol. 2023 May 22;3:1144162. doi: 10.3389/fepid.2023.1144162 (PMC10910898; doi:10.3389/fepid.2023.1144162)
Supplement: Supplementary file 1 [file Datasheet1.docx]

***SUPPLEMENTARY MATERIAL***

**Cross-sectional and longitudinal comparison of health-related quality of life and mental well-being between persons with and without post COVID-19 condition**

**Emily Stella Scott^1^*, Erica I. Lubetkin^2^, Mathieu F. Janssen^3^, John Yfantopolous^4^, Gouke J. Bonsel^5^, Juanita A. Haagsma^1^**

*** Correspondence:** Emily Stella Scott [e.scott@erasmusmc.nl](mailto:e.scott@erasmusmc.nl)

**Table S1. Disease status categorisation and their definitions in 2020 (T1) and 2022 (T2), n=4999**

| 2020 (T1) | | | 2022 (T2) | | |
| --- | --- | --- | --- | --- | --- |
| Disease status | **Definition** | N (%) | **Disease status** | **Definition** | N (%) |
| *Healthy* | No chronic condition(s)  No acute COVID-19 infection  No post COVID-19 condition | 2696 (54%) | *Healthy* | No chronic condition(s)  No acute COVID-19 infection  No post COVID-19 condition  Never a COVID-19 infection  Recovered from past likely or confirmed COVID-19 infection  Acute COVID-19 infection within the past 3 months prior to the questionnaire but no symptoms | 2854 (57%) |
| *Chronic condition(s)* | One or more chronic condition(s)  No post COVID-19 condition  No acute COVID-19 infection | 1980 (40%) | *Chronic condition(s)* | One or more chronic condition(s)  No post COVID-19 condition  No acute COVID-19 infection | 1798 (36%) |
| *Possible acute or past COVID-19 infection* | Likely COVID-19 infection due to slight fever or coughing symptoms. Has not been tested and does not receive any special care (248 responses)  Likely COVID-19 infection due to symptoms or a positive test (66)  Likely COVID-19 infection and still experiencing moderate to severe health problems (9) | 323 (6%) | *Acute COVID-19 infection* | Likely or confirmed COVID-19 infection sometime within the last 3 months prior to the questionnaire  Indicating COVID-19 symptoms | 107 (2%) |
|  |  |  | *Post COVID-19 condition* | Likely or confirmed COVID-19 infection sometime more than 3 months ago prior to the questionnaire  Indicating still suffering from COVID-19 symptoms | 240 (5%) |

**Table S2. Non-response analysis of the dropout in 2020 (T1) vs respondents in 2022 (T2), at T1**

|  |  | Dropout after T1 (n=14903) | Completer T1 and T2 (n=4999) | p-value |
| --- | --- | --- | --- | --- |
| Gender | Male | 6925 (46.5) | 2369 (47.4) | .009 |
|  | Female | 7939 (53.3) | 2627 (52.6) |  |
|  | Other | 39 (0.3) | 3 (0.1) |  |
| Age | Median (IQR) | 43 (26) | 53 (22) | <.001 |
|  | Mean (SD) | 44.2 (16) | 51.8 (13.6) |  |
| Age category | 18 – 24 | 1814 (12.2) | 132 (2.6) | <.001 |
|  | 25 – 34 | 3065 (20.6) | 514 (10.3) |  |
|  | 35 – 44 | 3093 (20.8) | 926 (18.5) |  |
|  | 45 – 54 | 2618 (17.6) | 1102 (22.0) |  |
|  | 55 – 64 | 2124 (14.3) | 1213 (24.3) |  |
|  | 65 – 75 | 2189 (14.7) | 1112 (22.2) |  |
| Educational level | High | 7853 (52.7) | 2534 (50.7) | .002 |
|  | Middle | 5478 (36.8) | 1973 (39.5) |  |
|  | Low | 1572 (10.5) | 492 (9.8) |  |
| Country | Greece | 646 (4.3) | 376 (7.5) | <.001 |
|  | Italy | 2047 (13.7) | 1165 (23.3) |  |
|  | The Netherlands | 2652 (17.8) | 644 (12.9) |  |
|  | Sweden | 2490 (16.7) | 729 (14.6) |  |
|  | United Kingdom | 2361 (15.8) | 873 (17.5) |  |
|  | United States | 4707 (31.6) | 1212 (24.2) |  |
| Chronic condition(s) | None | 7760 (52.1) | 2825 (56.5) | <.001 |
|  | One or more | 7143 (47.9) | 2174 (43.5) |  |
| Disease status | Healthy | 7176 (48.2) | 2854 (57.1) | NA |
|  | Chronic condition(s) | 6059 (40.7) | 1798 (36) |  |
|  | Possible acute or past COVID-19 infection | 1668 (11.2) | NA |  |
|  | Acute COVID-19 infection | NA | 107 (2.1) |  |
|  | Post COVID-19 condition | NA | 240 (4.8) |  |


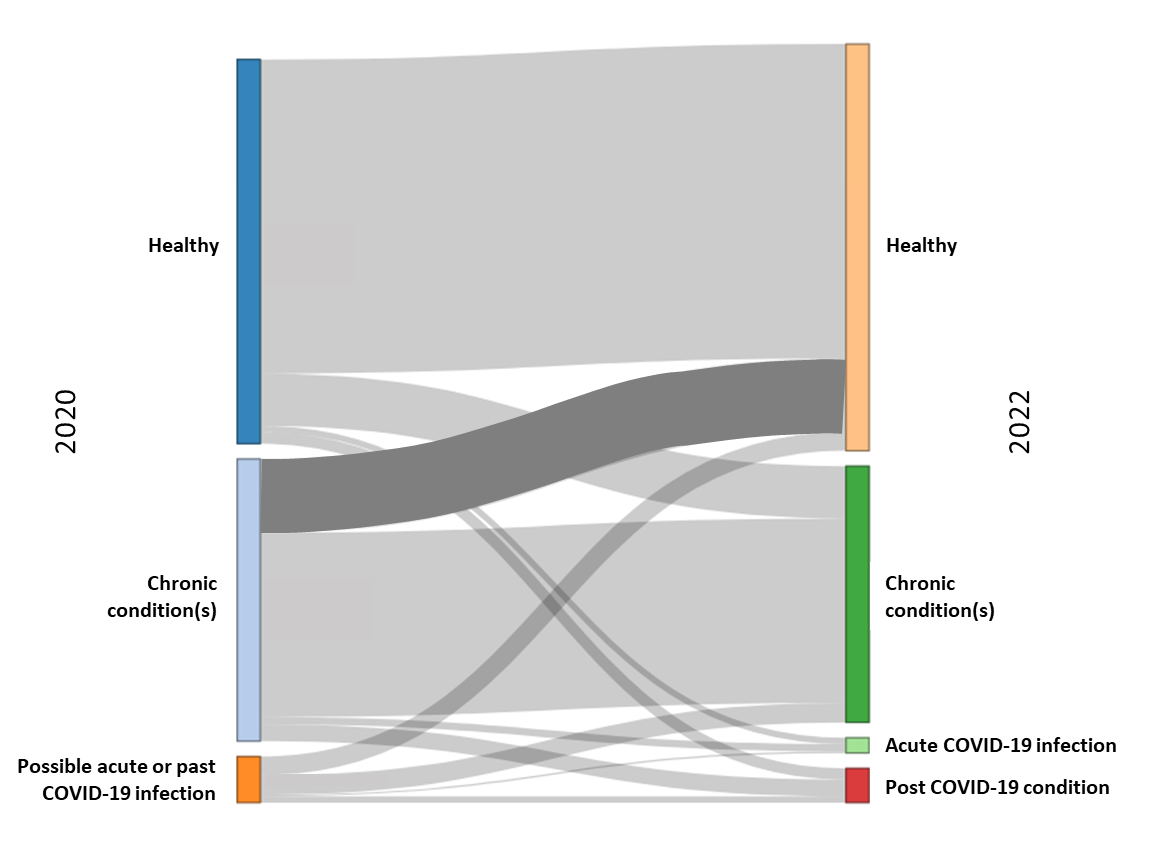


**Figure S1. Sankey plot of the transitions between disease status groups in 2020 (T1) (Healthy, Chronic condition(s), Possible acute or past COVID-19 infection) to 2022 (T2) (Healthy, Chronic condition(s), Acute COVID-19 infection and Post COVID-19 condition)***

*Further analyses were conducted for the number of participants with chronic condition(s) at T1 becoming healthy at T2, for which the group is highlighted in the figure. There were 517 (18.1% of those within the healthy group at T2) within this category, compared to the number of participants transitioning from healthy at T1 to chronic conditions at T2 (367 (20.4% of those within the chronic condition(s) group at T2). Upon this, the types of chronic conditions in the participants that transitioned from chronic to healthy were thoroughly analysed, showing that they were largely symptom treatable/manageable or indeed curable.

**Figure S2(A). Percentage of respondents per level per EQ-5D-5L dimension domain in 2020** **(T1) and 2022 (T2), by T2 disease status, for those aged 18 – 54**

**Figure S2(B). Percentage of respondents per level per EQ-5D-5L dimension domain in 2020 (T1) and 2022 (T2), by T2 disease status, for those aged 55 – 77**

**Figure S3(A). Percentage of respondents with poor mental health according to the WHO-5, PHQ-9 and GAD-7 in 2020 (T1) and 2022 (T2), by T2 disease status, for those aged 18 – 54**

**Figure S3(B). Percentage of respondents with poor mental health according to the WHO-5, PHQ-9 and GAD-7 stacked bar charts by disease status in 2020 and 2022, by T2 disease status for those aged 55 – 77**

**Table S3. Mean and median EQ-5D-5L index value, EQ VAS, GAD-7 sum score, PHQ-9 sum score and WHO-5 sum score in 2020 (T1) and 2022 (T2), by T2 disease status**

|  |  | Disease status | | | | | | | |  | |
| --- | --- | --- | --- | --- | --- | --- | --- | --- | --- | --- | --- |
| Outcome variable | **Statistic** | **Healthy** | | **Chronic condition(s)** | | **Acute COVID-19 infection** | | **Post COVID-19 condition** | | **p-value at T2** | |
|  |  | 2020 | 2022 | 2020 | 2022 | 2020 | 2022 | 2020 | 2022 |  |  |
| EQ-5D-5L index value | Mean (SD) | 0.91 (0.13) | 0.92 (0.12) | 0.78 (0.24) | 0.75 (0.25) | 0.75 (0.31) | 0.73 (0.29) | 0.72 (0.29) | 0.70 (0.29) | <.001 |  |
|  | Median (IQR) | 0.94 (0.12) | 0.94 (0.12) | 0.87 (0.25) | 0.82 (0.29) | 0.88 (0.35) | 0.84 (0.32) | 0.82 (0.35) | 0.78 (0.28) |  |  |
|  | Min. – Max. | -0.16 – 1 | -0.45 – 1 | -0.57 – 1 | -0.49 – 1 | -0.35 – 1 | -0.22 – 1 | -0.42 – 1 | -0.22 – 1 |  |  |
| EQ VAS | Mean (SD) | 82.2 (13.5) | 80.8 (14.3) | 71.7 (19.3) | 68.4 (19.8) | 71.4 (21.5) | 65.1 (20.1) | 70.6 (20.4) | 65.7 (21.0) | <.001 |  |
|  | Median (IQR) | 85 (10) | 80 (15) | 80 (25) | 70 (20) | 80 (30) | 70 (30) | 75 (25) | 70 (29) |  |  |
|  | Min. – Max. | 0 – 100 | 5 – 100 | 0 – 100 | 0 – 100 | 20 – 100 | 15 – 95 | 0 – 100 | 0 – 100 |  |  |
| WHO-5 sum | Mean (SD) | 66 (22.4) | 65.6 (23.2) | 58.6 (24.6) | 56.1 (25.9) | 55.9 (25.5) | 51.1 (25.6) | 50.2 (25.3) | 47.7 (26.7) | <.001 |  |
|  | Median (IQR) | 72 (28) | 72 (28) | 64 (40) | 60 (44) | 60 (44) | 56 (40) | 50 (44) | 44 (48) |  |  |
|  | Min. – Max. | 0 – 100 | 0 – 100 | 0 – 100 | 0 – 100 | 8 – 100 | 0 – 100 | 0 – 100 | 0 – 100 |  |  |
| PHQ-9 sum | Mean (SD) | 3.6 (4.4) | 2.8 (4.0) | 5.4 (5.6) | 5.2 (5.6) | 6.5 (6.1) | 7.7 (6.5) | 8.7 (7.0) | 9.1 (6.7) | <.001 |  |
|  | Median (IQR) | 2 (5) | 1 (4) | 4 (7) | 3 (7) | 5 (7) | 6 (10) | 7 (11) | 8 (11) |  |  |
|  | Min. – Max. | 0 – 27 | 0 – 27 | 0 – 27 | 0 – 27 | 0 – 27 | 0 – 25 | 0 – 27 | 0 – 26 |  |  |
| GAD-7 sum | Mean (SD) | 3.6 (4.3) | 2.5 (3.8) | 4.7 (4.9) | 4.2 (4.9) | 5.5 (5.4) | 5.7 (5.7) | 7.6 (6.0) | 7.4 (5.8) | <.001 |  |
|  | Median (IQR) | 2 (6) | 1 (4) | 4 (7) | 3 (6) | 5 (6) | 4 (8) | 7 (9) | 7 (8) |  |  |
|  | Min. – Max. | 0 – 21 | 0 – 21 | 0 – 21 | 0 – 21 | 0 – 21 | 0 – 21 | 0 – 21 | 0 – 21 |  |  |

EQ-5D-5L index values range from less than 0 (worse than death) to 1. The EQ VAS (Visual analogue scale) ranges from 0 (worst self-rated health) to 100 (best self-rated health). SD = Standard deviation. IQR = Interquartile range. The WHO-5 sum score (WHO-5 Well-being index) ranges from 0 (worst imaginable well-being) to 100 (best imaginable well-being). The PHQ-9 sum score (Patient health questionnaire 9) ranges from 0 to 27 (Mild: 5-9, Moderate: 10-14, Moderately severe: 15-19, Severe: 20-27). The GAD-7 sum score (General anxiety disorder 7-item scale) ranges from 0 to 21 (Mild: 5-9, Moderate: 10-14, Severe: 15-21).

**Table S4. Multiple comparisons post-hoc analysis test of the one-way ANOVA in 2022 (T2), using the Bonferroni correction**

| Dependent Variable | (A) Disease status at T2 | (B) Disease status at T2 | Mean Difference (A-B) | Std. Error | Sig. | 95% Confidence Interval | |
| --- | --- | --- | --- | --- | --- | --- | --- |
|  |  |  |  |  |  | *Lower Bound* | *Upper Bound* |
| EQ-5D-5L index value | Healthy | Chronic condition(s) | .166* | 0.006 | <.001 | 0.150 | 0.181 |
|  | Healthy | Acute COVID-19 infection | .184* | 0.019 | <.001 | 0.134 | 0.234 |
|  | Healthy | Post COVID-19 condition | .220* | 0.013 | <.001 | 0.185 | 0.254 |
|  | Chronic condition(s) | Acute COVID-19 infection | 0.018 | 0.019 | 1 | -0.032 | 0.069 |
|  | Chronic condition(s) | Post COVID-19 condition | .054* | 0.013 | <.001 | 0.019 | 0.089 |
|  | Acute COVID-19 infection | Post COVID-19 condition | 0.035 | 0.022 | 0.693 | -0.024 | 0.095 |
| EQ VAS score | Healthy | Chronic condition(s) | 12.349* | 0.510 | <.001 | 11.000 | 13.690 |
|  | Healthy | Acute COVID-19 infection | 15.704* | 1.668 | <.001 | 11.300 | 20.100 |
|  | Healthy | Post COVID-19 condition | 15.104* | 1.138 | <.001 | 12.100 | 18.110 |
|  | Chronic condition(s) | Acute COVID-19 infection | 3.355 | 1.685 | 0.279 | -1.090 | 7.800 |
|  | Chronic condition(s) | Post COVID-19 condition | 2.755 | 1.164 | 0.108 | -0.320 | 5.830 |
|  | Acute COVID-19 infection | Post COVID-19 condition | -0.6 | 1.969 | 1 | -5.800 | 4.600 |
| WHO-5 sum score | Healthy | Chronic condition(s) | 9.490* | 0.735 | <.001 | 7.550 | 11.430 |
|  | Healthy | Acute COVID-19 infection | 14.480* | 2.404 | <.001 | 8.140 | 20.830 |
|  | Healthy | Post COVID-19 condition | 17.896* | 1.641 | <.001 | 13.570 | 22.230 |
|  | Chronic condition(s) | Acute COVID-19 infection | 4.99 | 2.429 | 0.24 | -1.420 | 11.400 |
|  | Chronic condition(s) | Post COVID-19 condition | 8.406* | 1.678 | <.001 | 3.980 | 12.830 |
|  | Acute COVID-19 infection | Post COVID-19 condition | 3.415 | 2.838 | 1 | -4.080 | 10.910 |
| PHQ-9 sum score | Healthy | Chronic condition(s) | -2.413* | 0.146 | <.001 | -2.800 | -2.030 |
|  | Healthy | Acute COVID-19 infection | -4.907* | 0.478 | <.001 | -6.170 | -3.650 |
|  | Healthy | Post COVID-19 condition | -6.293* | 0.326 | <.001 | -7.150 | -5.430 |
|  | Chronic condition(s) | Acute COVID-19 infection | -2.494* | 0.483 | <.001 | -3.770 | -1.220 |
|  | Chronic condition(s) | Post COVID-19 condition | -3.881* | 0.334 | <.001 | -4.760 | -3.000 |
|  | Acute COVID-19 infection | Post COVID-19 condition | -1.387 | 0.564 | 0.084 | -2.880 | 0.100 |
| GAD-7 sum score | Healthy | Chronic condition(s) | -1.637* | 0.132 | <.001 | -1.980 | -1.290 |
|  | Healthy | Acute COVID-19 infection | -3.162* | 0.430 | <.001 | -4.300 | -2.030 |
|  | Healthy | Post COVID-19 condition | -4.889* | 0.294 | <.001 | -5.670 | -4.110 |
|  | Chronic condition(s) | Acute COVID-19 infection | -1.525* | 0.435 | 0.003 | -2.670 | -0.380 |
|  | Chronic condition(s) | Post COVID-19 condition | -3.252* | 0.300 | <.001 | -4.050 | -2.460 |
|  | Acute COVID-19 infection | Post COVID-19 condition | -1.727* | 0.508 | 0.004 | -3.070 | -0.390 |

* The mean difference is significant at the 0.05 level.

**Table S5. Spearman’s rank correlation analysis of the EQ-5D-5L utility, EQ VAS, WHO-5, PHQ-9 and GAD-7 sum scores by disease status at T2, in 2020 (T1) and 2022 (T2)**

| Year | Disease status at T2 | Outcome measure | EQ-5D-5L index value | EQ VAS score | WHO-5 sum score | PHQ-9 sum score | GAD-7 sum score |
| --- | --- | --- | --- | --- | --- | --- | --- |
| *2020* | *Healthy* | **EQ-5D-5L index value** | 1 | .444* | .463* | -.522* | -.493* |
|  |  | **EQ VAS score** | .444* | 1 | .468* | -.413* | -.359* |
|  |  | **WHO-5 sum score** | .463* | .468* | 1 | -.713* | -.660* |
|  |  | **PHQ-9 sum score** | -.522* | -.413* | -.713* | 1 | .802* |
|  |  | **GAD-7 sum score** | -.493* | -.359* | -.660* | .802* | 1 |
|  | *Chronic condition(s)* | **EQ-5D-5L index value** | 1 | .610* | .501* | -.548* | -.459* |
|  |  | **EQ VAS score** | .610* | 1 | .489* | -.447* | -.335* |
|  |  | **WHO-5 sum score** | .501* | .489* | 1 | -.749* | -.682* |
|  |  | **PHQ-9 sum score** | -.548* | -.447* | -.749* | 1 | .807* |
|  |  | **GAD-7 sum score** | -.459* | -.335* | -.682* | .807* | 1 |
|  | *Acute COVID-19 infection* | **EQ-5D-5L index value** | 1 | .662* | .571* | -.570* | -.458* |
|  |  | **EQ VAS score** | .662* | 1 | .564* | -.536* | -.400* |
|  |  | **WHO-5 sum score** | .571* | .564* | 1 | -.773* | -.727* |
|  |  | **PHQ-9 sum score** | -.570* | -.536* | -.773* | 1 | .855* |
|  |  | **GAD-7 sum score** | -.458* | -.400* | -.727* | .855* | 1 |
|  | *Post COVID-19 condition* | **EQ-5D-5L index value** | 1 | .665* | .540* | -.596* | -.452* |
|  |  | **EQ VAS score** | .665* | 1 | .532* | -.475* | -.319* |
|  |  | **WHO-5 sum score** | .540* | .532* | 1 | -.702* | -.612* |
|  |  | **PHQ-9 sum score** | -.596* | -.475* | -.702* | 1 | .845* |
|  |  | **GAD-7 sum score** | -.452* | -.319* | -.612* | .845* | 1 |
| *2022* | *Healthy* | **EQ-5D-5L index value** | 1 | .426* | .442* | -.528* | -.479* |
|  |  | **EQ VAS score** | .426* | 1 | .482* | -.392* | -.348* |
|  |  | **WHO-5 sum score** | .442* | .482* | 1 | -.660* | -.628* |
|  |  | **PHQ-9 sum score** | -.528* | -.392* | -.660* | 1 | .787* |
|  |  | **GAD-7 sum score** | -.479* | -.348* | -.628* | .787* | 1 |
|  | *Chronic condition(s)* | **EQ-5D-5L index value** | 1 | .602* | .499* | -.543* | -.447* |
|  |  | **EQ VAS score** | .602* | 1 | .502* | -.447* | -.313* |
|  |  | **WHO-5 sum score** | .499* | .502* | 1 | -.738* | -.676* |
|  |  | **PHQ-9 sum score** | -.543* | -.447* | -.738* | 1 | .807* |
|  |  | **GAD-7 sum score** | -.447* | -.313* | -.676* | .807* | 1 |
|  | *Acute COVID-19 infection* | **EQ-5D-5L index value** | 1 | .632* | .586* | -.594* | -.528* |
|  |  | **EQ VAS score** | .632* | 1 | .479* | -.461* | -.434* |
|  |  | **WHO-5 sum score** | .586* | .479* | 1 | -.733* | -.703* |
|  |  | **PHQ-9 sum score** | -.594* | -.461* | -.733* | 1 | .775* |
|  |  | **GAD-7 sum score** | -.528* | -.434* | -.703* | .775* | 1 |
|  | *Post COVID-19 condition* | **EQ-5D-5L index value** | 1 | .634* | .535* | -.623* | -.527* |
|  |  | **EQ VAS score** | .634* | 1 | .483* | -.518* | -.426* |
|  |  | **WHO-5 sum score** | .535* | .483* | 1 | -.637* | -.609* |
|  |  | **PHQ-9 sum score** | -.623* | -.518* | -.637* | 1 | .838* |
|  |  | **GAD-7 sum score** | -.527* | -.426* | -.609* | .838* | 1 |

* Correlation is significant at the 0.01 level (2-tailed).
